# Device-Less Data-Driven Cardiac and Respiratory Gating Using LAFOV PET Histo Images

**DOI:** 10.3390/diagnostics14182055

**Published:** 2024-09-16

**Authors:** Nanna Overbeck, Thomas Lund Andersen, Anders Bertil Rodell, Jorge Cabello, Noah Birge, Paul Schleyer, Maurizio Conti, Kirsten Korsholm, Barbara Malene Fischer, Flemming Littrup Andersen, Ulrich Lindberg

**Affiliations:** 1 Department of Clinical Physiology and Nuclear Medicine, Copenhagen University Hospital—Rigshospitalet, 2100 Copenhagen, Denmark; thomas.lund.andersen@regionh.dk (T.L.A.); kirsten.korsholm.01@regionh.dk (K.K.); barbara.malene.fischer@regionh.dk (B.M.F.); flemming.andersen@regionh.dk (F.L.A.); ulrich.lindberg@regionh.dk (U.L.); 2 Department of Clinical Medicine, Copenhagen University, 2100 Copenhagen, Denmark; 3 Siemens Healthcare A/S, 8200 Aarhus, Denmark; anders.rodell@siemens-healthineers.com; 4 Siemens Medical Solutions USA, Inc., Knoxville, TN 37932, USA; jorge.cabello@siemens-healthineers.com (J.C.); noah.birge@siemens-healthineers.com (N.B.); paul.schleyer@siemens-healthineers.com (P.S.); maurizioconti@siemens-healthineers.com (M.C.)

**Keywords:** data-driven gating, image-derived frequency, gating, motion correction, respiration, cardiac, histo images, TOF

## Abstract

**Background:** The outstanding capabilities of modern Positron Emission Tomography (PET) to highlight small tumor lesions and provide pathological function assessment are at peril from image quality degradation caused by respiratory and cardiac motion. However, the advent of the long axial field-of-view (LAFOV) scanners with increased sensitivity, alongside the precise time-of-flight (TOF) of modern PET systems, enables the acquisition of ultrafast time resolution images, which can be used for estimating and correcting the cyclic motion. **Methods:** 0.25 s so-called [^18^F]FDG PET histo image series were generated in the scope of for detecting respiratory and cardiac frequency estimates applicable for performing device-less data-driven gated image reconstructions. The frequencies of the cardiac and respiratory motion were estimated for 18 patients using Short Time Fourier Transform (STFT) with 20 s and 30 s window segments, respectively. **Results:** The Fourier analysis provided time points usable as input to the gated reconstruction based on eight equally spaced time gates. The cardiac investigations showed estimates in accordance with the measured pulse oximeter references (*p* = 0.97) and a mean absolute difference of 0.4 ± 0.3 beats per minute (bpm). The respiratory frequencies were within the expected range of 10–20 respirations per minute (rpm) in 16 out of 18 patients. Using this setup, the analysis of three patients with visible lung tumors showed an increase in tumor SUV_max_ and a decrease in tumor volume compared to the non-gated reconstructed image. **Conclusions:** The method can provide signals that were applicable for gated reconstruction of both cardiac and respiratory motion, providing a potential increased diagnostic accuracy.

## 1. Introduction

Positron Emission Tomography (PET) combined with Computed Tomography (CT) is a valuable tool for diagnosis and treatment planning in oncology, neurology, and cardiology [1]. PET is a molecular imaging technique for mapping physiological processes. The conventional PET/CT system includes a field-of-view (FOV) of 15–30 cm. To cover the whole body, multiple bed positions are necessary. This causes a trade-off between the number of detected coincidence events of each position, the scanning duration, and the injected radioactivity. The development of long axial field-of-view (LAFOV) PET/CT systems allows scanning of the entire thorax in a single bed position alongside the benefit of an improvement in sensitivity since fewer photons escape the detector field [2]. This sensitivity increase can be effectively translated to decreased dose or scan time [3] but also gives the opportunity to acquire a PET dynamic time series of sub-second timing resolution.

Respiratory motion of the patient’s chest during a PET scan acquisition leads to decreased image quality due to blurring. Such blurring furthers a decrease in tumor maximum and mean Standardized Uptake Value (SUV_max_, SUV_mean_), increased detected tumor volume, and a potential decrease in clinical lesion detectability. Furthermore, the mismatch between CT and PET due to movement can cause incorrect attenuation correction of the reconstructed PET image [4,5,6,7].

Cardiac contractions can also contribute to additional image blurring alongside the respiratory movement. This is a problem since cardiac investigations such as myocardial perfusion examinations and myocardial vitality investigations depend highly on accurate relative quantification of tracer distribution in the heart [8]. The quantification is also sensitive to incorrect attenuation of the PET image from the mismatch of the CT density image to the moving heart. Overall, movement during PET examination can lead to incorrect staging and diagnosis as well as mislocated lesion detection, which can cause radiation dose escalation due to mislocated therapy planning [9].

A known method for alleviating the problems of motion is by gating the reconstructed images. Respiratory-gated images have shown improvement regarding image quality in the thorax region [10], whereas cardiac-gated images are applicable for the calculation of clinical parameters such as ejection fraction [11]. Gating provides motion-corrected images based on the division of emission data into selected periods (gates). Different methodologies have been investigated for gating in the last decades [12]. External devices are commonly used for motion tracking of the respiratory cycle or measuring cardiac rhythm, such as a pressure belt positioned on the patient’s abdomen or using an electrocardiogram (ECG) integrated with the PET/CT system. The use of external devices demands additional time and inconvenience during patient preparation and adds tasks for the scanner personnel [13,14,15,16].

In contrast to device-driven gating, device-less data-driven gating facilitates an easier clinical routine. Moreover, it has also been shown that data-driven gating performs superiorly to gating based on external devices [17,18]. Previously investigated methods for data-driven motion estimation include, among others, Fourier frequency analysis/spectral analysis, combining signal fluctuations, the sensitive method, the center-of-mass method, and/or Principal Component Analysis (PCA) [8,16,17,19,20,21,22,23,24,25]. The studies of Schleyer et al. (2009, 2011) present a data-driven gating method using Fourier frequency analysis for multibed position PET/CT. In this study, they obtained the respiratory signal based on masks within sinogram space, and the respiratory trace was compared to external device measurements, suggesting that Fourier analysis of the masked sinogram data provides information valid for respiratory gating [19,26]. Another study investigating device-less data-driven gating is Thielemans et al. (2014), which uses a generated dynamic series of 0.2 s frames for extraction of the respiratory and cardiac signal in the scope of performing dual gating. The group concludes that the respiratory signal was in accordance with the measured reference; however, they were not able to derive a high-quality cardiac signal for 2-deoxy-2-[^18^F]fluoro-D-glucose ([^18^F]FDG) studies [27].

In the current study, we present the use of histo images for device-less data-driven gating of [^18^F]FDG PET images. Histo images are generated images based on the time-of-flight (TOF) information obtained during the scan acquisition [28,29,30]. The simplest method for generating PET histo images is by using the maximum likelihood position (MLP) of the annihilation event position using the TOF information [30]. Yusheng Li et al. (2019) have investigated the use of PET histo images for motion correction using an XCAT cardiac phantom, and their motion field estimation is used for correcting cardiac motion [31]. In addition, the study of Büther et al. (2020) presented the use of histo images for performing respiratory-gated reconstructions determining the anterior–posterior distribution in counts for mapping the initial respiratory amplitude [32].

To our knowledge, the use of a LAFOV PET histo image series for estimation of both respiratory and cardiac frequency and the application of output results for gated image reconstructions have not yet been evaluated. This study aims to evaluate the use of a high-sensitivity LAFOV PET histo image series in the scope of obtaining a device-less data-driven frequency estimate of the cardiac and respiratory frequencies. Furthermore, it is aimed to provide gated PET image reconstructions based on the frequency estimate outputs.

## 2. Materials and Methods

### 2.1. Patients

The patients included in this study were scanned approximately one hour p.i. of 3 MBq per kg body weight (223 MBq ± 65 MBq) [^18^F]FDG using a LAFOV PET/CT system (Siemens Biograph Vision Quadra, Siemens Healthineers, Erlangen, Germany), including 106 cm within the one-bed position. In total, 18 patients (13 females, 5 males) at the age of 59.4 ± 14.9 years were included in this study. The patients were scanned from October 2023 to May 2024. Patients 1–15 were prospective and randomly selected from the clinical routine of the department. The PET scan acquisitions were used for proof-of-concept investigations. Patients 16–18 were referred to the department due to a suspected tumor located in the lungs. These patients were selected in the scope of investigating respiratory affected tumors in gated image reconstructions compared to the reconstructed image with free breathing, of which the latter is termed the non-gated reconstructions in this paper.

All patients were scanned for five minutes. The scans were performed head first with arms up, except in one case, where the patient only raised one arm. A pulse oximeter (PM10N, Nellcor, Medtronic, Minneapolis, MN, USA) with ±2% accuracy was attached to the patient’s index finger during the full scan procedure. The measurements of the pulse oximeter were used as a cardiac frequency reference. Data from the pulse oximeter were exported using the Nellcor Analytics Tool.

The patient data were treated anonymously in accordance with the European General Data Protection Regulation (GDPR), and the patients gave informed consent to participate in the study, which was approved by the departmental review board (no. 576_23).

### 2.2. Data-Driven Frequency Estimation

The dynamic histo image series were generated using an algorithm integrated with an investigational software prototype (e7tools, VR20b, Siemens Healthineers, Erlangen, Germany). Based on MLP, the histogramming tool provided histo images with a defined matrix size of 220 × 220 × 645, corresponding to a voxel size of 3.3 × 3.3 × 1.65 mm^3^. The dynamic histo image series were generated with a temporal resolution of 0.25 s (4 Hz), leading to 1200 image frames for a PET scan of five minutes.

Using the TotalSegmentator algorithm (version 2), CT-based masks of the left ventricle and the lower lobe of the right lung were obtained [33]. The CT-based segmentation masks were used to extract the average PET time activity signal from the respective organs.

The frequency estimates were obtained by performing Short Time Fourier Transform (STFT) using a Hann window. The cardiac frequency estimate was obtained using a 20 s window. The window was shifted by 75% between each analyzed segment in accordance with Constant-Overlap-Add (COLA). The respiratory frequency estimate was obtained using a 30 s window with a corresponding segment shift of 75% overlap. STFT provided a spectrogram in which the maximum amplitude in a predefined frequency range was assumed to be the physiological movement frequencies. The dominant cardiac frequencies were extracted with a lower threshold of 30 beats per minute (bpm) and an upper threshold of 120 bpm defined from the chosen sample frequency of 4 Hz. The respiratory rate was found using a lower threshold of 9 respiration per minute (rpm) and an upper threshold of 24 rpm. The analysis of the signals was performed using the Python SciPy package (version 1.14.0).

### 2.3. Gated Image Reconstruction

The signal of the STFT was filtered by frequency masking, providing a signal of the frequency traces containing the filtered estimates that show frequencies within the restricted frequency band of the analysis. The frequency masking of the cardiac signal was the detected dominant frequency of the spectral analysis ±0.25 Hz (±15 bpm). Correspondingly, the frequency masking of the respiratory signal was performed using the dominant frequency ±0.10 Hz (±6 rpm). The inverse STFT of the respiratory signal provided a time domain signal applicable for respiratory-gated image reconstruction. The time points of each period of the respiratory signal were found and used for the gated reconstruction.

The extracted average time activity curve of the left ventricle was used as the cardiac signal in the gated image reconstruction. Segments of 20 s of the cardiac signal were transformed using the Fast Fourier transform (FFT). For each cardiac signal segment, the dominant frequency and the corresponding phase of the signal were found based on the FFT spectral analysis. The resultant frequency and phase were used for reconstructing a time signal, enabling the selection of time points for each period used during the image-gated reconstruction process. The listmode data were divided into eight sinograms, followed by the reconstruction, leading to eight final reconstructed images. The division of the data was chosen to be eight equally sized segments throughout the cardiac cycle corresponding to a phase-based gating.

Reconstructions of non-gated and gated PET images were performed using 3D Ordinary Poisson Ordered Subset Expectation Maximization with Point Spread Function modeling (PSF-OP-OSEM) with 4 iterations, 5 subsets, and a maximum ring difference (MRD) of 322. A Gaussian post-filtering was performed with 2 mm FWHM, resulting in a matrix size of 440 × 440 and a 1.65 mm slice thickness. The reconstructions were all performed offline using investigational software prototypes (e7tools version VR20, Siemens Healthineers).

### 2.4. PET Image Evaluation

The output trace of the cardiac frequency signal was evaluated by visualizing the trace for all patients, along with the respective reference measurement signal obtained by pulse oximeter. Furthermore, the mean value of the five-minute scan was compared to the reference measurements using an unpaired two-sample *t*-test using the null hypothesis of the mean values being equal.

The mean respiratory frequency estimate was evaluated relative to the known range of normal breathing (10–20 rpm) [9]. Furthermore, the gated reconstruction was demonstrated by the gated reconstructed images reflecting the breathing cycle. Patients 16–18 with a tumor located in the lung were analyzed relative to the tumor SUV_max_, SUV_mean_, and the tumor volume using a Syngo.Via workstation (Siemens Healthineers AG, Erlangen, Germany). The tumor region was found using an isocontour of 40% of the maximum uptake. The tumor volume and SUV values were compared to a non-gated reconstruction of the patient scan.

## 3. Results

**Figure 1:**
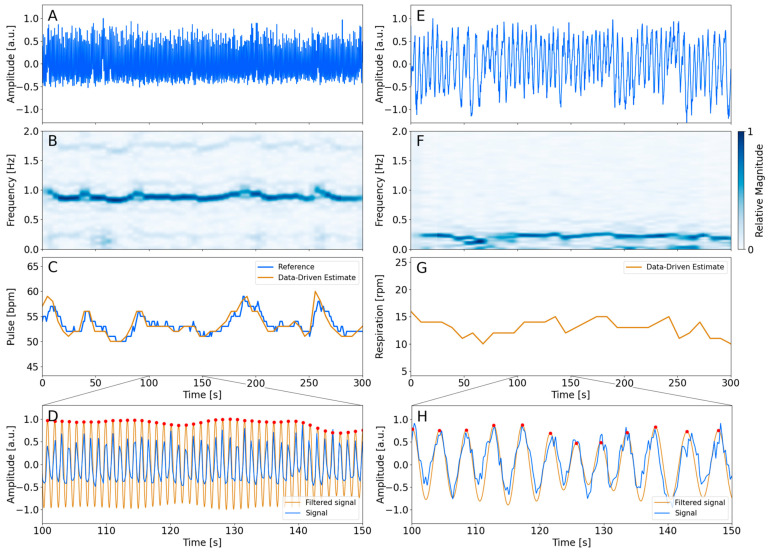
Overview of the processing steps of the analysis. The left column represents the cardiac analysis of patient 9. The right column represents the respiratory analysis of the same patient. The average time activity signal from the left ventricle and right lung is visualized in the top row (**A**,**E**). (**B**,**F**) show the spectrogram found using STFT, of which the signal with the largest magnitude is shown as a 2D plot in (**C**,**G**). The filtered inverse STFT signal is visualized in (**D**,**H**) along with the average time activity signal of the masks. The red dots indicate the period of each cycle. The time points were used for the respiratory-gated reconstruction.

The procedure for obtaining the cardiac (Figure 1A–D) and respiratory (Figure 1E–H) trace is shown in Figure 1. Figure 1A,E show the average signal obtained using the masks of the left ventricle and the right lung, respectively. The analysis of each signal provides a spectrogram shown in Figure 1B,F. The observed maximum magnitude of the spectrum corresponded to the estimate of the frequency traces. The cardiac and respiratory traces are visualized in Figure 1C,G. The cardiac trace is visualized along with the reference measurements. The figure indicates an agreement between the device-less data-driven cardiac estimate and the measured pulse reference. The respiratory trace fluctuates within the expected range. Figure 1D,H show the filtered signal from the inverse STFT in the range of 100 s and 150 s of the acquisition.

Table 1 summarizes the estimated mean cardiac and respiratory frequency throughout the acquired five minutes. Furthermore, the measured cardiac reference frequency is listed as well. The table indicates that the data-driven estimated cardiac frequency is in accordance with the reference measurements. The absolute difference between the estimated data-driven frequencies and the reference frequencies was calculated to be less than a maximum of 1.4 bpm, and the overall mean difference between the data-driven estimates and the reference measurements was found to be 0.4±0.3 bpm. A two-sampled *t*-test supports the interpretation that the two methods agreed due to the failure of rejecting the null hypothesis (*p* = 0.97). The estimated respiratory frequency of the patients shows a mean value between 10 and 20 rpm in 16 out of 18 patient investigations. One patient shows a lower respiration rate than 10 rpm, and a single patient shows a respiratory rate higher than 20 rpm. The average respiration rate was found to be 14.1±3.1.

**Figure 2:**
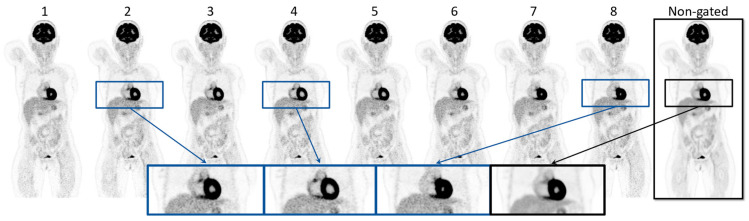
Illustration of the gate 1–8 of the gated reconstructed images of patient 16 along with the non-gated reconstruction of the same PET acquisition. The zoomed images visualize gates 2, 4, and 8 in the cardiac cycle, along with the non-gated image.

**Figure 3:**
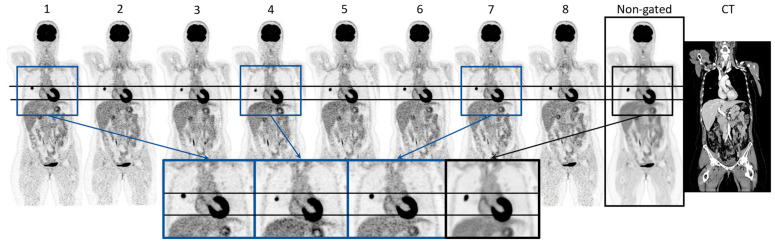
Visualization of the respiratory gated reconstruction (gate 1–8) containing, along with the non-gated reconstruction image and CT of patient 16. The horizontal lines indicate the top position of the tumor and the liver referenced at gate 1. The zoomed images of gates 1, 4, and 7, and the non-gated image highlight the differences in the position and appearance of the tumor and the liver.

Figure 2 visualizes the eight gates obtained through the cardiac-gated image reconstruction of patient 16 along with the non-gated image reconstruction. Visual inspection showed that the volume of the heart increases and decreases throughout the eight cardiac gates in accordance with a cardiac cycle. This is highlighted by the enlarged images of the heart. The same patient is shown in Figure 3, illustrating the respiratory-gated image reconstructions with a moving lung tumor throughout the respiratory cycle. The non-gated reconstruction is visualized as well, showing a smearing of the tumor. The highlighted enlarged images of the lung region in Figure 3 visualize the displacement of the tumor relative to a reference line. See Supplementary Videos S1 and S2 for an animation of the cardiac- and respiratory-gated reconstructions. The SUV_max_, SUV_mean_, and the volume of the lung tumors of patients 16–18 are summarized in Table 2. SUV_max_ and SUV_mean_ were increased in all cases relative to the SUV_max_ and SUV_mean_ of the tumor estimated based on the non-gated reconstructed image, except for a single gate of patient 18. The gate showing the largest differences from the non-gated reconstructed image is highlighted in blue in Table 2. The investigations show that the gated image reconstruction provides uptake values of tumor SUV_max_ and SUV_mean_ that are 67.3%, 17.1%, and 58.3% larger than the tumor SUV of the non-gated image, respectively. Furthermore, the volume of the tumor was found to decrease by −41.7%, −29.0%, and −64.3%, respectively.

## 4. Discussion

This study presented a proof-of-concept method for device-less data-driven LAFOV PET histo image-based respiratory- and cardiac-gated PET image reconstruction.

The findings of the study showed that the extracted signals of the left ventricle and right lung from the sub-second LAFOV PET histo image series were a feasible tool for providing an accurate estimate of the respiratory and cardiac frequency using a standard clinical [^18^F]FDG protocol both prospectively and retrospectively. The data-driven estimated mean cardiac frequencies were found to be in accordance with the measured reference signal of the pulse oximeter, supported by the findings of small absolute differences, which were summarized in Table 1. The result of this study presented a method for accurate and robust cardiac frequency estimation.

The method enabled visualization of the cardiac contractions and depicted a clear difference in the lumen volume within the myocardium, suggesting that the analysis provided accurate time points for cardiac gated reconstructions (Figure 2). The visual appearance of the heart tissue was clearly defined and much improved when comparing the different gated images to the non-gated images.

Studies have investigated the use of data-driven methods for performing gating of physiological movement. Most studies presented methods for correcting respiratory motion, as this contributes to large displacements during scan acquisition. Wang et al. (2015) and Schleyer et al. (2011) generated 0.1 s sinogram frames for obtaining a frequency estimate of the respiratory waveform [14,23]. Visvikis et al. (2004) investigated respiratory gating using 0.15 s, 0.45 s, and 0.65 s frames, and Thielemans et al. (2014) used 0.2 s frames with their method for performing dual gating [24,26]. The use of sub-second signals was thus found to be a necessity to evaluate a well-sampled movement. This study presents a 0.25 s frame duration corresponding to a 4 Hz sampling frequency. This was a tradeoff between sampling fast enough to capture most cardiac frequencies and maintaining an adequate signal for the device-less data-driven cardiac extraction. This assumption was in line with our pulse oximeter measurements. Even though one patient showed a cardiac frequency in the near range of the limitation (120 bpm), it was possible to determine a robust data-driven cardiac frequency estimate (Patient 2). A higher temporal resolution was possible and could be utilized to capture higher cardiac frequencies if needed; however, it was hypothesized that most patients would have a cardiac frequency below 120 bpm after a bed rest of one hour between injection and scanning.

The method for estimating the cardiac and respiratory frequency in this paper was performed using the STFT. Fourier frequency analysis is a common tool for the estimation of the dominant frequencies of other studies. Schleyer et al. (2013) utilized STFT for data-driven respiratory gating parallel to tracer kinetics investigations. The group experienced that a sign correction was a necessity for obtaining a respiratory signal in agreement with the respiratory motion reference signal [18]. The use of STFT for mapping the respiratory signal was successful in this study; however, the inverse STFT of the cardiac signal showed inaccuracies regarding the time determination of the cycle. Regular FFT provided sufficient frequency and phase estimates of the window durations to obtain satisfying timing definitions that are usable for performing the gated image reconstruction. Other groups have obtained their respiratory or motion signal using regions of interest, edge boxes, or center-of-mass methods. Furthermore, multiple groups filter their signal using PCA [34]. The study by Wang et al. (2015) explained a comparison of using either the center-of-mass method or PCA for extraction of the respiratory signal relative to an external device. As their investigation was performed using 0.1 s PET images, the SNR is relatively low. Within these conditions, the group concludes that center-of-mass performed superiorly using a single examination, thus implying additional investigations are necessary [24]. The analysis of the patients included in this study showed promising results without using PCA for feature extraction and instead focusing on the signal extracted from the organ masks. The risk of overfitting the output signal using STFT due to the presence of noise was decreased by applying a limited band relative to the overall found frequency estimate of either the respiratory or cardiac movement.

An important aspect of clinical decision-making is accurate [^18^F]FDG uptake quantification and lesion volume estimation. For example, measurements of metabolic tumor volume or total lesion glycolysis have shown predictive value in lung cancer [35]. It is well-known that respiratory motion affects SUV measurements and volume estimates. The respiratory-gated sequences in this study showed expected results with an SUV increase, e.g., from an SUV of 10.1 to 16.8. Vicente et al. (2011) showed respiratory gating led to an increase in SUV_max_ in 54 out of 57 patients investigated [6]. Furthermore, the study described that the gated image reconstructions could visualize more lesions than the non-gated reconstructed PET image. The study by Noto et al. (2022) utilized a data-driven gating method to demonstrate SUV differences between non-gated reconstructed images compared to gated image reconstructions using their data-driven method but also using respiratory belt trigger signals. Their data-driven method performed favorably relative to the use of an external device [36]. The respiratory-gated reconstruction shown in Figure 3 highlighted the effect of performing the gating of motion-affected tumors. Both the tumor and the liver displacement could influence the interpretation of the PET image. The majority of the eight gated images of each of the patients with a tumor located in the lung showed an increase in the SUV_max_, SUV_mean,_ and a decrease in tumor volume, indicating that the non-gated reconstruction provides a smeared-out expression of the tumor. This corresponded to the expectations found in the literature. The gating of the five-minute PET acquisition caused the gated images to contain coincidence events corresponding to approximately 1/8 of the 300 s collected data. This is of sub-clinical PET quality but might be sufficient to achieve an accurate lesion measurement. The change in SUV_max_ observed between each gated image was likely affected by a change in signal-to-noise ratio (SNR). Additionally, a co-registration of the gated images was not performed in this study, which would have resulted in a SNR of the gated images comparable to the non-gated PET image. The relative differences between the tumor SUVs of the gated images and the non-gated image of patient 17 were of smaller magnitude as the tumor was positioned in the top right lobe of the lung. Small increases in SUVs were, however, observed, highlighting the influence of respiratory movement on lung tumor quantification, even in low displacement areas of the lung. The application of respiratory gating of patient scans with lung tumors has high clinical value within radiation therapy planning. This field is especially dependent on the quantitation of the tumor regions. In the daily clinical routine across all types of oncological examinations, the PET investigation is performed using free breathing. This may be due to the lack of effective data-driven tools [37]. It is of interest to evaluate this method regarding its application within radiation therapy with the potential of more accurate dose planning using device-less data-driven gating.

The main limitation of this study was the lack of a respiratory reference signal and a reference signal for both the cardiac and respiratory motion incorporated into the scanner system, thus providing the gold standard for the gating procedure. The study by Walker et al. (2020) presented a comparison of their device-less data-driven method relative to gated reconstruction using an external device. Their findings showed that using an external device failed in 16% of the examinations. The remaining investigations showed preferable results using their device-less method in 13% of the cases and preferable results using external devices in 2% of the cases. Otherwise, the two methods performed equivalent [38]. The equivalent performance of the two procedures contributed to the benefits of using a device-less method during the clinical routine, as the resulting reconstruction was equivalent, and the additional setup during the patient examination was avoided.

This proof-of-concept study was sufficient to provide findings robustly stating the dominant frequencies of the cardiac and respiratory rates using the cohort size of 18 patients. The study illustrated its potential for clinical application within respiratory gating of patients with lung tumors; however, a limitation was that only three patients included possessed a lung tumor.

The range of methods for obtaining the respiratory frequency signal or the cardiac contraction rate is shown by multiple groups, highlighting that this area is undergoing constant development. It is believed that the novel method presented in this study provides sufficient and robust results using a method with great potential due to the aspect of generating an automated procedure for obtaining gated PET reconstructed images relative to organ-specific movement directly in image space.

It was observed that the cardiac signal contained less noise if there was a clear uptake of the tracer in the myocardium. This was not always the case, as the myocardium might utilize energy derived from free fatty acids rather than from glucose [39]. This study was based solely on patient scans using [^18^F]FDG. Initial work included the investigation of dynamic scans using ^15^O-labelled water ([^15^O]H_2_O). The study showed a high influence of the bolus injection; however, it was possible to accurately estimate the cardiac frequency of the patients. It is of interest to evaluate the robustness of this method regarding other types of tracers, which may or may not show uptake within the myocardium.

The method was found to be robust relative to larger fluctuations of the cardiac frequency throughout the PET acquisition time. One patient showing a higher degree of fluctuations was visualized in Figure 1. Generally, it was found that the estimated data-driven cardiac traces were in accordance with the measured reference by following the fluctuations throughout the five minutes. This supports the interpretation of the robustness of the method.

The presented method provided motion-compensated images. The use of a device-less data-driven gating approach enables the gating of more patient scans since it alleviates the setup time of hardware for recording a respiratory or cardiac signal. Furthermore, this methodology enables a retrospective analysis of patients showing signs of cardiac disease as a response to cancer treatment. Future investigations include the expansion of this proof-of-concept study to include a larger cohort size. Additionally, the aim is to validate the method by obtaining an image quality assessment by an experienced MD. Other future investigations are to examine clinical applications of this method, especially regarding cardiac function, such as the retrieval of the clinical ejection fraction.

## 5. Conclusions

The use of a LAFOV PET histo image series was found to be a feasible method for device-less data-driven frequency estimation of cardiac and respiratory movement during PET scan acquisition in clinical routine. The fully data-driven method showed accurate cardiac estimates in comparison to reference measurements. Additionally, data-driven respiratory frequency estimates provided reasonable results within an expected range. The gated image reconstructions showed isolation of the heart and respiratory movement. The respiratory-gated images provided tumor quantification with an increase in SUV_max_ and SUV_mean_, as well as a decrease in the measured tumor volume.

## Figures and Tables

**Table 1:** Overview of the mean and standard deviation $\left(\mu \pm \sigma \right)$ frequency estimate of the data-driven cardiac estimate, cardiac reference measurement, and the data-driven respiratory estimate. The bottom row summarizes the overall mean and standard deviation of the respective column across all patients.

Patient Number	Mean Data-Driven Cardiac Estimate $\left(\mu \pm \sigma \right)$ [bpm]	Mean Cardiac Reference Measurement $\left(\mu \pm \sigma \right)$ [bpm]	Absolute Difference [bpm]	Mean Data-Driven Respiratory Estimate $\left(\mu \pm \sigma \right)$ [rpm]
1	58.9±1.3	58.6±1.1	0.3	12.2±1.6
2	108.5±3.1	109.0±1.7	0.5	13.3±2.5
3	54.7±1.3	55.0±1.1	0.3	14.6±1.5
4	70.5±1.3	70.4±1.1	0.1	13.9±1.9
5	78.3±1.7	78.3±1.6	0.0	13.1±1.9
6	74.4±2.4	74.0±2.5	0.4	9.0±1.0
7	70.6±1.2	70.7±1.1	0.1	17.6±0.5
8	85.1±1.4	85.4±1.4	0.3	16.1±1.5
9	53.4±2.4	53.4±1.9	0.0	12.8±2.2
10	92.3±2.2	92.2±1.4	0.1	14.4±1.9
11	57.4±3.8	58.1±0.8	0.7	18.9±1.4
12	43.7±9.2	45.0±0.7	1.3	14.8±1.4
13	85.0±2.5	86.1±2.1	1.1	15.0±1.6
14	59.4±1.1	59.1±0.7	0.3	12.5±1.5
15	57.9±2.0	58.2±1.5	0.3	21.5±1.6
16	74.8±2.0	74.6±2.1	0.2	10.4±4.6
17	94.0±1.5	94.1±0.8	0.1	11.8±1.9
18	72.1±4.8	72.7±3.6	0.6	11.0±3.6
Overall	71.9±16.3	71.7±16.4	0.4±0.3	14.1±3.1

**Table 2:** Overview of SUV_max_, SUV_mean_, and the tumor volume, based on a 40% isocontour, for the respiratory-gated reconstruction of patients 16, 17, and 18, together with the values of the tumors based on a non-gated reconstruction. The gate showing the largest difference to the non-gated reconstructed image is marked in blue, and the non-gated image values are marked in red. The relative difference in percent is calculated based on the marked gate for each patient using the non-gated values as reference $\left(\frac{gated-non\text{-}gated}{non\text{-}gated}\times 100\%\right)$.

	Patient 16			Patient 17			Patient 18		
	Max [SUV]	Mean [SUV]	Volume [cm^3^]	Max [SUV]	Mean [SUV]	Volume [cm^3^]	Max [SUV]	Mean [SUV]	Volume [cm^3^]
**1**	16.8	10.5	1.0	17.8	9.5	8.8	3.5	1.9	0.5
**2**	14.3	8.8	1.1	16.8	9.4	10.1	2.4	1.4	1.1
**3**	12.7	8.0	1.3	16.3	9.2	11.1	2.2	1.2	1.3
**4**	13.9	9.0	1.1	17.5	9.5	9.4	2.6	1.4	0.8
**5**	13.3	8.2	1.3	17.5	9.5	9.4	2.0	1.2	1.6
**6**	14.9	9.4	1.1	16.7	9.3	10.9	2.5	1.4	1.1
**7**	15.6	10.1	1.1	17.0	9.4	9.9	2.4	1.3	1.1
**8**	16.9	10.5	1.0	17.0	9.4	9.2	3.1	1.7	0.7
**Non-gated**	10.1	6.4	1.7	15.2	8.7	12.4	2.1	1.2	1.4
**Max rel. diff.**	67.3%	64.1%	−41.2%	17.1%	9.2%	−29.0%	66.7%	58.3%	−64.3%

## Data Availability

Data supporting reported results can be obtained via contact with the corresponding author upon reasonable request and legal approval. The data are not publicly available due to a no public data sharing agreement.

## Supplementary Materials

The following supporting information can be downloaded at: https://www.mdpi.com/article/10.3390/diagnostics14182055/s1, Video S1. Video of the cardiac movement; Video S2. Video of the respiratory movement.

## References

[1] Hervás Morón A. (2007). PET-CT in oncology. Clin. Transl. Oncol..

[2] Prenosil G.A., Sari H., Fürstner M., Afshar-Oromieh A., Shi K., Rominger A., Hentschel M. (2022). Performance Characteristics of the Biograph Vision Quadra PET/CT System with a Long Axial Field of View Using the NEMA NU 2-2018 Standard. J. Nucl. Med..

[3] Calderón E., Schmidt F.P., Lan W., Castaneda-Vega S., Brendlin A.S., Trautwein N.F., Dittmann H., la Fougère C., Kiefer L.S. (2023). Image Quality and Quantitative PET Parameters of Low-Dose [18F]FDG PET in a Long Axial Field-of-View PET/CT Scanner. Diagnostics.

[4] Harteela M., Hirvi H., Mäkipää A., Teuho J., Koivumäki T., Mäkelä M.M., Teräs M. (2014). Comparison of end-expiratory respiratory gating methods for PET/CT. Acta Oncol..

[5] Kang S.Y., Moon B.S., Kim H.O., Yoon H.J., Kim B.S. (2021). The impact of data-driven respiratory gating in clinical F-18 FDG PET/CT: Comparison of free breathing and deep-expiration breath-hold CT protocol. Ann. Nucl. Med..

[6] Vicente A.M.G., Castrejón A.S., Martín A.A.L., García B.G., Woll J.P.P., Muñoz A.P. (2011). Value of 4-Dimensional 18F-FDG PET/CT in the Classification of pulmonary lesions. J. Nucl. Med. Technol..

[7] Guerra L., de Ponti E., Elisei F., Bettinardi V., Landoni C., Picchio M., Gilardi M.C., Versari A., Fioroni F., Dziuk M. (2012). Respiratory gated PET/CT in a European multicentre retrospective study: Added diagnostic value in detection and characterization of lung lesions. Eur. J. Nucl. Med. Mol. Imaging.

[8] Lassen M.L., Kwiecinski J., Slomka P.J. (2019). Gating Approaches in Cardiac PET Imaging. PET Clin..

[9] Lang N., Dawood M., Büther F., Schober O., Schäfers M., Schäfers K. (2006). Organ Movement Reduction in PET/CT using Dual-Gated Listmode Acquisition. Z. Med. Phys..

[10] Messerli M., Liberini V., Grünig H., Maurer A., Skawran S., Lohaus N., Husmann L., Orita E., Trinckauf J., Kaufmann P.A. (2021). Clinical evaluation of data-driven respiratory gating for PET/CT in an oncological cohort of 149 patients: Impact on image quality and patient management. Br. Inst. Radiol..

[11] Stegger L., Heijman E., Schäfers K.P., Nicolay K., Schäfers M.A., Strijkers G.J. (2009). Quantification of left ventricular volumes and ejection fraction in mice using PET, compared with MRI. J. Nucl. Med..

[12] Büther F., Ernst I., Dawood M., Kraxner P., Schäfers M., Schober O., Schäfers K.P. (2010). Detection of respiratory tumour motion using intrinsic list mode-driven gating in positron emission tomography. Eur. J. Nucl. Med. Mol. Imaging.

[13] Lupi A., Zaroccolo M., Salgarello M., Malfatti V., Zanco P. (2009). The effect of 18F-FDG-PET/CT respiratory gating on detected metabolic activity in lung lesions. Ann. Nucl. Med..

[14] Kesner A.L., Kuntner C. (2010). A new fast and fully automated software based algorithm for extracting respiratory signal from raw PET data and its comparison to other methods. Med. Phys..

[15] Shi C., Tang X., Chan M. (2017). Evaluation of the new respiratory gating system. Precis. Radiat. Oncol..

[16] Kesner A.L., Schleyer P.J., Büther F., Walter M.A., Schäfers K.P., Koo P.J. (2014). On transcending the impasse of respiratory motion correction applications in routine clinical imaging—A consideration of a fully automated data driven motion control framework. EJNMMI Phys..

[17] Büther F., Ernst I., Frohwein L.J., Pouw J., Schäfers K.P., Stegger L. (2018). Data-driven gating in PET: Influence of respiratory signal noise on motion resolution. Med. Phys..

[18] Schleyer P.J., Thielemans K., Marsden P.K. (2014). Extracting a Respiratory Signal from Raw Dynamic PET Data That Contain Tracer Kinetics. Phys. Med. Biol..

[19] Schleyer P.J., O’Doherty M.J., Marsden P.K. (2011). Extension of a data-driven gating technique to 3D, whole body PET studies. Phys. Med. Biol..

[20] Feng T., Yang G., Zhang X., Berg E., Liu H., Lv Y., Qi J., Cherry S.R., Badawi R.D. A Fast Local Gating Method for TOF-PET. Proceedings of the 2020 IEEE Nuclear Science Symposium and Medical Imaging Conference (NSS/MIC).

[21] Panin V.Y., Bal H. TOF Data Non-Rigid Motion Correction. Proceedings of the 2015 IEEE Nuclear Science Symposium and Medical Imaging Conference (NSS/MIC).

[22] Hwang D., Kang S.K., Kim K.Y., Choi H., Seo S., Lee J.S. (2021). Data-driven respiratory phase-matched PET attenuation correction without CT. Phys. Med. Biol..

[23] Armstrong I.S., Memmott M.J., Saint K.J., Saillant A., Hayden C., Arumugam P. (2021). Assessment of motion correction in dynamic rubidium-82 cardiac PET with and without frame-by-frame adjustment of attenuation maps for calculation of myocardial blood flow. J. Nucl. Cardiol..

[24] Wang M., Guo N., Zhang H., Elfhakri G., Hu G., Li Q. Retrospective data-driven respiratory gating for PET using TOF information. Proceedings of the 2015 37th Annual International Conference of the IEEE Engineering in Medicine and Biology Society (EMBC).

[25] Visvikis D., Barret O., Fryer T., Turzo A., Lamare F., Le Rest C.C., Bizais Y. A posteriori respiratory motion gating of dynamic PET images. Proceedings of the 2003 IEEE Nuclear Science Symposium.

[26] Schleyer P.J., O’Doherty M.J., Barrington S.F., Marsden P.K. (2009). Retrospective data-driven respiratory gating for PET/CT. Phys. Med. Biol..

[27] Thielemans K., Schleyer P., Marsden P.K., Teuho J., Teras M., Bettinardi V., Menezes L., Manjeshwar R.M., Stearns C.W. Data-driven Dual-gating for Cardiac PET. Proceedings of the 2014 IEEE Nuclear Science Symposium and Medical Imaging Conference (NSS/MIC).

[28] Vandenberghe S., Daube-Witherspoon M.E., Lewitt R.M., Karp J.S. (2006). Fast reconstruction of 3D time-of-flight PET data by axial rebinning and transverse mashing. Phys. Med. Biol..

[29] Daube-Witherspoon M.E., Matej S., Werner M.E., Surti S., Karp J.S. (2012). Comparison of list-mode and DIRECT approaches for time-of-flight PET reconstruction. IEEE Trans. Med. Imaging.

[30] Conti M. (2011). Focus on time-of-flight PET: The benefits of improved time resolution. Eur. J. Nucl. Med. Mol. Imaging.

[31] Li Y., Karp J.S., Matej S. Motion Displacement Field Estimation using Time-of-Flight PET Histoimages. Proceedings of the 2019 IEEE Nuclear Science Symposium and Medical Imaging Conference (NSS/MIC).

[32] Büther F., Jones J., Seifert R., Stegger L., Schleyer P., Schäfers M. (2020). Clinical evaluation of a data-driven respiratory gating algorithm for whole-body PET with continuous bed motion. J. Nucl. Med..

[33] Wasserthal J., Breit H.-C., Meyer M.T., Pradella M., Hinck D., Sauter A.W., Heye T., Boll D.T., Cyriac J., Yang S. (2023). TotalSegmentator: Robust Segmentation of 104 Anatomic Structures in CT Images. Radiol. Artif. Intell..

[34] Thielemans K., Rathore S., Engbrant F., Razifar P. Device-less gating for PET ICT using PCA. Proceedings of the 2011 IEEE Nuclear Science Symposium Conference Record.

[35] Wen W., Piao Y., Xu D., Li X. (2021). Prognostic Value of MTV and TLG of 18F-FDG PET in Patients with Stage i and II Non-Small-Cell Lung Cancer: A Meta-Analysis. Contrast Media Mol. Imaging.

[36] Noto B., Roll W., Zinken L., Rischen R., Kerschke L., Evers G., Heindel W., Schäfers M., Büther F. (2022). Respiratory motion correction in F-18-FDG PET/CT impacts lymph node assessment in lung cancer patients. EJNMMI Res..

[37] Zhong H., Ren L., Lu Y., Liu Y. (2023). On the correction of respiratory motion-induced image reconstruction errors in positron-emission tomography-guided radiation therapy. Phys. Imaging Radiat. Oncol..

[38] Walker M.D., Morgan A.J., Bradley K.M., McGowan D.R. (2020). Data-driven respiratory gating outperforms device-based gating for clinical 18F-FDG PET/CT. J. Nucl. Med..

[39] Minamimoto R. (2021). Series of myocardial FDG uptake requiring considerations of myocardial abnormalities in FDG-PET/CT. Jpn. J. Radiol..

